# Directly Observed Therapy Reduces Tuberculosis-Specific Mortality: A Population-Based Follow-Up Study in Taipei, Taiwan

**DOI:** 10.1371/journal.pone.0079644

**Published:** 2013-11-22

**Authors:** Yung-Feng Yen, Muh-Yong Yen, Yi-Ping Lin, Hsiu-Chen Shih, Lan-Huei Li, Pesus Chou, Chung-Yeh Deng

**Affiliations:** 1 Section of Infectious Diseases, Taipei City Hospital, Taipei City Government, Taipei, Taiwan; 2 Department of Disease Control and Prevention, Taipei City Hospital, Taipei City Government, Taipei, Taiwan; 3 Taipei Databank for Public Health Analysis, Taipei City Hospital, Taipei City Government, Taipei, Taiwan; 4 Community Medicine Research Center and Institute of Public Health, National Yang-Ming University, Taipei, Taiwan; 5 Institute of Hospital and Health Care Administration, National Yang-Ming University, Taipei, Taiwan; National Institute of Allergy and Infectious Diseases, United States of America

## Abstract

**Objectives:**

To determine the effect of directly observed therapy (DOT) on tuberculosis-specific mortality and non-TB-specific mortality and identify prognostic factors associated with mortality among adults with culture-positive pulmonary TB (PTB).

**Methods:**

All adult Taiwanese with PTB in Taipei, Taiwan were included in a retrospective cohort study in 2006–2010. Backward stepwise multinomial logistic regression was used to identify risk factors associated with each mortality outcome.

**Results:**

Mean age of the 3,487 patients was 64.2 years and 70.4% were male. Among 2471 patients on DOT, 4.2% (105) died of TB-specific causes and 15.4% (381) died of non-TB-specific causes. Among 1016 patients on SAT, 4.4% (45) died of TB-specific causes and 11.8% (120) died of non-TB-specific causes. , After adjustment for potential confounders, the odds ratio for TB-specific mortality was 0.45 (95% CI: 0.30–0.69) among patients treated with DOT as compared with those on self-administered treatment. Independent predictors of TB-specific and non-TB-specific mortality included older age (ie, 65–79 and ≥80 years vs. 18–49 years), being unemployed, a positive sputum smear for acid-fast bacilli, and TB notification from a general ward or intensive care unit (reference: outpatient services). Male sex, end-stage renal disease requiring dialysis, malignancy, and pleural effusion on chest radiography were associated with increased risk of non-TB-specific mortality, while presence of lung cavities on chest radiography was associated with lower risk.

**Conclusions:**

DOT reduced TB-specific mortality by 55% among patients with PTB, after controlling for confounders. DOT should be given to all TB patients to further reduce TB-specific mortality.

## Introduction

Tuberculosis (TB) is a deadly infectious disease that has been prevalent throughout human history. To improve treatment adherence among TB patients, in 2005, the World Health Organization (WHO) issued a recommendation for directly observed therapy, short course (DOTS) [1]. As of 2009, more than 149 countries had adopted DOTS, and 61.7% (5.8 million) of the 9.4 million incident TB cases were treated using the DOTS approach [2]. More importantly, the WHO-recommended target of 85% treatment—ie, 85% of patients receiving TB treatment are cured or completely treated—was first attained in 2005 and was achieved again each year from 2007 through 2009 [2].

In Taiwan, TB has for decades been the most commonly reported infectious disease [3]. In 2010, 13,336 new TB cases were reported to the Taiwan Centers for Disease Control (CDC): 1.1% were multidrug-resistant TB (MDR-TB) and 95.5% were pulmonary TB (PTB) [3]. To achieve a treatment success rate of 85% and halve TB incidence by 2015, the Taiwan CDC adopted the DOTS protocol in 2006 [3]. Since then, TB incidence decreased from 72.0 per 100,000 in 2005 to 57.8 in 2009, while the treatment success rate increased from 64.1% to 69.9% during the same period [3].

DOT was designed to encourage treatment adherence and completion, thereby reducing mortality and preventing drug resistance. However, a 2009 Cochrane review of DOT trials concluded that the effects of DOT on reducing all-cause mortality and achieving treatment success among TB patients were similar to those of self-administered treatment (SAT) [4]. However, the sample of DOT trials that were considered may not have been representative of the true population as patients with severe illness, multidrug-resistant TB, and those with relapse of TB were excluded [5]–[8]. Moreover, a trial [8] reported that when the families of TB patients served as treatment observers, they failed to follow WHO guidelines, which yielded lower cure rates and much higher default rates than those obtained with observers trained by the relevant health care system [9]–[11]. An additional two trials found that treatment outcome did not significantly differ between SAT and DOT when supervised by observers trained by health care professionals. However, the sample sizes of those studies were small [7], [12].

According to the WHO, death—defined as a patient who dies for any reason during treatment—is an unacceptable outcome of TB treatment [13]. However, many TB patients die of other causes, eg, cancer or end-stage renal disease (ESRD). A recent review article found that few studies of TB treatment outcomes distinguished between TB-specific and non-TB-specific mortality [14]. Thus this study was aimed to investigate factors associated with TB-specific and non-TB-specific mortality, respectively, among Taiwanese adults with culture-positive pulmonary TB (PTB) during the period 2006–2010. We hypothesized that after accounting for confounding variables, patients on DOT would have a lower risk of TB-specific mortality than those on SAT.

## Methods

### Study setting and population

This retrospective cohort study used TB surveillance data collected by the Taipei city government in Taiwan. The subjects included in this study were Taiwanese adults (age ≥18 years) with culture-positive PTB in Taipei during the period 2006–2010. , This research was approved by the Institutional Review Board of Taipei City Hospitals. The patients' written consents were waived by the approving IRB because personally identifying information were not included in the dataset.

### Data collection and DOT

TB is reportable disease in Taiwan. When TB cases were reported to Taipei TB Control Department, trained case managers used a structured questionnaire to interview patients about their sociodemographic characteristics (age, sex, marital status, education level, nursing home residence, smoking, alcohol use, and being unemployed), underlying diseases (malignancy, ESRD requiring dialysis), admission history, and TB treatments. Clinical examinations (including chest radiography, acid-fast bacilli [AFB] smear status, drug resistance) were provided by reporting hospitals.

Taiwan CDC began to implement the DOTS strategy from 2006. Although DOT was not compulsory to TB patients, Taiwan CDC strongly recommended it as the optimal treatment strategy, especially for TB patients at a great risk for transmitting TB (eg, patients with positive AFB smear) or for TB treatment nonadherence and failure (eg, relapsing cases) [3]. When patients agreed to DOT, a trained supervisor would observe them taking medications (5 times a week at a minimum). Patients on DOT in Taiwan were given adherence-promoting incentives, including social service support and food coupons equal to US$2/day. Those not assigned to DOT or that refused DOT were assigned to SAT.

Both the TB patients on DOT and those on SAT were followed up regularly by cases managers in person or by telephone until treatment success, treatment default, treatment failure, transfer, or death, as defined by WHO criteria [15]. The follow-up was once every other week in the first month of TB treatment and once a month afterwards. In each visit, case managers interviewed the patients regarding treatment complications and if needed, provided them with social support [3].

### Outcome variables

The outcome variable of interest was treatment outcome, which was categorized into three groups: successful treatment (ie, cure or completed treatment) [15], TB-specific death, and non-TB-specific death For the purpose of TB control, Taipei TB Control Department worked with Taiwan Department of Health for data linkage in order to identify the information of cause of death for every TB patient with death in Taipei TB datasets [16]. In Taiwan it is regulated by the law that within 30 days after a patient dies, his or her death certificate must be issued and registered by the physician in charge according to International Classification of Diseases (ICD) 9 or 10. Trained medical registrars review and code all death certificates at the central office of the National Death Certification Registry. As a result, the cause-of-death coding in Taiwan has been considered very accurate [17]. TB-specific death in this study was defined as the underlying cause of death due to pulmonary TB in Taiwan Death Certification Registry (A011 in ICD-9 and A162 in ICD-10). Non-TB-specific death was defined as any underlying cause of death other than TB. The successful treatment group was used as the reference.

### Main explanatory variable

The main explanatory variable was mode of TB treatment (DOT vs. SAT). DOT took place at either the patients' residence or workplace. DOT was defined as antituberculosis medication ingestion directly supervised by trained public health observers instead of by family members [18], [19]. SAT was used to refer to any unsupervised treatment.

### Control variables

Control variables included patient sociodemographics, clinical findings, underlying diseases, and source of TB notification. Education level was categorized as uneducated, elementary school, high school, and university or higher. Unemployment was defined as being unemployed on the date of TB notification. The reporting source was defined as the department that reported the TB case, namely, a general ward, intensive care unit (ICU), or outpatient service.

### Statistical analysis

Since this study is aimed to evaluate the effect of DOT on mortality, PTB patients who were lost to follow up during treatment were excluded from this analysis. Also, this analysis excluded TB patients with missing data about marital status, unemployment, smoking and alcohol use, because these cases did not account for TB-specific or non-TB-specific deaths.

Descriptive statistics were used to characterize patients by treatment modality. In bivariate analysis, logistic regression was used to assess associations of selected factors with each outcome. All variables found to be statistically significant (*P*<0.10) in bivariate analysis were considered for inclusion in backward stepwise multinomial regression analysis. Since treatment mode (DOT vs. SAT) is the main explanatory variable, this factor was kept enforcedly in the final multinomial regression analysis. Adjusted odds ratios (AORs) and 95% confidence intervals (95% CIs) are reported as measures of association. All analyses were done with SPSS version 21.0 statistical software (SPSS, Chicago IL, USA).

## Results

**Figure 1:**
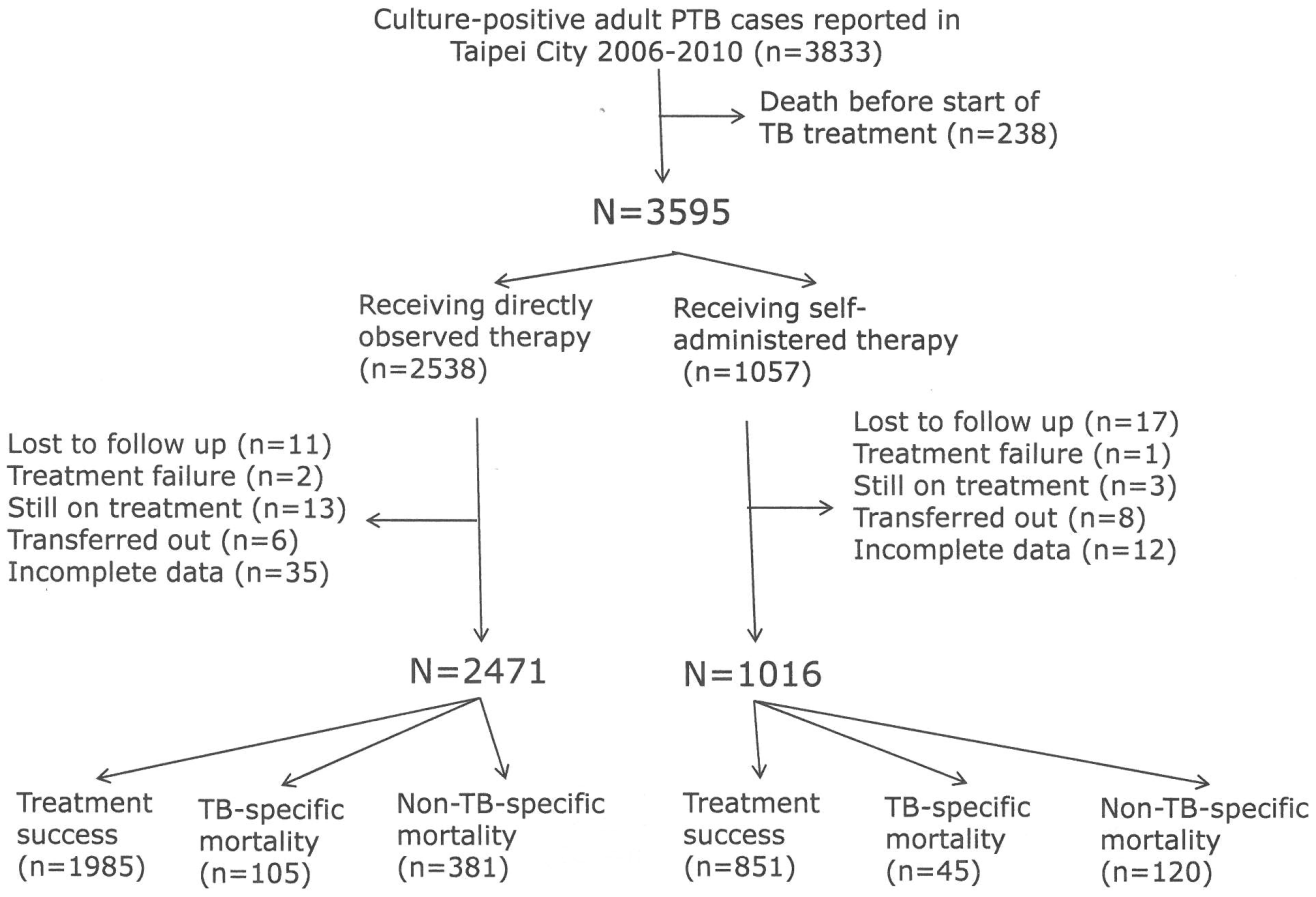
Study flow diagram. PTB, pulmonary tuberculosis.

During the 5-year follow-up, there were 3,833 adult (age ≥18 years) culture-positive PTB cases reported to the Taipei TB Control Department (Figure 1). Of these patients, 238 died before starting TB treatment and 3595 received treatment. Among the 3595 patients, 70.6% (2538) received DOT and 29.4% (1057) received SAT. After excluding that 28 were lost to follow-up during TB treatment, 16 were still receiving treatment at the time of this study, 3 experienced treatment failure, 14 had transferred out of Taipei city, and 47 had incomplete data, the remaining 2471 on DOT and 1016 on SAT were included in subsequent analysis. Overall mean age was 64.2 years (range 18–98 years), 70.4% were male. Among the 2471 patients on DOT, 4.2% (105) died of TB-specific causes and 15.4% (381) died of non-TB-specific causes. Also, among the 1016 patients on SAT, 4.4% (45) died of TB-specific causes and 11.8% (120) died of non-TB-specific causes. Among the 3487 cases in this analysis, the 3 most common causes of non-TB-specific mortality were cardiovascular diseases (88; 17.6%), malignancy (81; 16.2%), and renal/liver failure (34; 6.8%). Also, among the 2471 patients on DOT, the 3 most common causes of non-TB-specific mortality were cardiovascular diseases (68; 17.8%), malignancy (63; 16.5%), and renal/liver failure (27; 7.1%).

The proportion of patients receiving DOT increased from 50.3% in 2006, to 78.2% in 2008, and to 81.4% in 2010 (p<.001), while the proportion of TB-specific deaths decreased from 5.8% in 2006, to 5.1% in 2008, and to 4.4% in 2010 (p = .053). The proportion of non-TB-specific deaths fluctuated, from 13.1% in 2006, up to 15.6% in 2007, down to 14.4% in 2009, and up to 16.8% in 2010.

Table 1 showed that compared with patients receiving SAT, patients on DOT were more likely to be older, unemployed, positive in the sputum smears, and with cavity lesion on CXR.

**Table 1:** Sociodemographic and clinical characteristics of adult PTB patients treated by directly observed therapy compared with those under self-administered therapy, Taipei, Taiwan, 2006–2010.

Characteristics	Self-Administered Therapy (N = 1016)	Directly Observed Therapy (N = 2471)	P value
	n (%)	n (%)	
Mean age at diagnosis, year (SD)	62.1 (20.1)	65.0 (20.2)	<.001
Male gender	684 (67.3)	1770 (71.6)	0.011
Unmarried status	202 (19.9)	480 (19.4)	0.757
Education level			
Not educated	56 (5.5)	197 (8.0)	<.001
Elementary school	191 (18.8)	570 (23.1)	
High school	328 (32.3)	873 (35.3)	
University or above	329 (32.4)	573 (23.2)	
Unknown	112 (11.0)	258 (10.4)	
Unemployment	673 (66.2)	1826 (73.9)	<.001
Nursing home resident	38 (3.7)	111 (4.5)	0.318
Current smoking			
No	877 (86.3)	1949 (78.9)	<.001
1–10 cigarettes/day	50 (4.9)	178 (7.2)	
>10 cigarettes/day	89 (8.8)	344 (13.9)	
Any alcohol use	68 (6.7)	253 (10.2)	0.001
ESRD receiving dialysis	23 (2.3)	48 (1.9)	0.542
Malignancy	69 (6.8)	174 (7.0)	0.792
Prior treatment of TB	17 (1.7)	69 (2.8)	0.053
Positive AFB smear	259 (25.5)	1504 (60.9)	<.001
Cavity on CXR	184 (18.1)	555 (22.5)	0.004
Pleural effusion on CXR	72 (7.1)	160 (6.5)	0.510
MDR-TB	10 (1.0)	22 (0.9)	0.792
Source of notification			
Outpatient services	658 (64.8)	1265 (51.2)	<.001
Ordinary ward	323 (31.8)	1120 (45.3)	
Intensive care unit	35 (3.4)	86 (3.5)	

TB, Tuberculosis; SD, standard deviation; ESRD, end-stage renal disease; AFB, acid-fast bacilli; CXR, chest X ray; MDR, multidrug resistant.

As shown in Table 2, TB-specific and non-TB-specific mortality among patients significantly increased as age increased (p<.001). Also, prognostic factors associated with TB-specific and non-TB-specific mortality included age 50–64, 65–79, and ≥80 years (reference: age 18–49 years), male sex, unemployed status, residence in a nursing home, and TB notification from a general ward or ICU (reference: outpatient service). A positive AFB smear was associated with TB-specific mortality, while ESRD requiring dialysis, malignancy, pleural effusion on chest radiography, and DOT were associated with non-TB-specific mortality. Variables associated with lower risk of TB-specific and non-TB-specific mortality included unmarried status and a high school or university or higher education (reference: uneducated). Moreover, any alcohol use and presence of lung cavities on chest radiography were associated with lower risk of non-TB-specific mortality.

**Table 2:** Sociodemographic and clinical findings by treatment outcome among adult PTB patients in Taipei, Taiwan, in 2006–2010.

	Treatment success† (N = 2836)	TB-specific deaths (N = 150)		Non-TB-specific deaths (N = 501)	
Factors	N (%)	N (%)	OR (95% CI)	N (%)	OR (95% CI)
Mode of treatment					
SAT	851 (30.0)	45 (30.0)	1	120 (24.0)	1
DOT	1985 (70.0)	105 (70.0)	1.001 (0.70–1.43)	381 (76.0)	1.36 (1.09–1.70)**
Age (years)					
18–49	823 (29.0)	8 (5.3)	1	17 (3.4)	1
50–64	613 (21.6)	11 (7.3)	1.85 (0.74–4.62)	50 (10.0)	3.95 (2.26–6.91)***
65–79	773 (27.3)	35 (23.3	4.66 (2.15–10.10)***	165 (32.9)	10.33 (6.21–17.19)***
≥80	627 (22.1)	96 (64.0)	15.75 (7.60–32.64)***	269 (53.7)	20.77 (12.58–34.28)***
Gender					
Female	899 (31.7)	33 (22.0)	1	101 (20.2)	1
Male	1937 (68.3)	117 (78.0)	1.65 (1.11–2.44)*	400 (79.8)	1.84 (1.46–2.32)***
Marital status					
Married	2218 (78.2)	129 (86.0)	1	458 (91.4)	1
Unmarried	618 (21.8)	21 (14.0)	0.58 (0.37–0.94)*	43 (8.6)	0.34 (0.24–0.47)***
Education level					
Not educated	174 (6.1)	18 (12.0)	1	61 (12.2)	1
Elementary school	585 (20.6)	37 (24.7)	0.61 (0.34–1.10)	139 (27.7)	0.68 (0.48–0.96)*
High school	1018 (35.9)	43 (28.7)	0.41 (0.23–0.72)**	140 (27.9)	0.39 (0.28–0.55)***
University or above	794 (28.0)	23 (15.3)	0.28 (0.15–0.53)***	85 (17.0)	0.31 (0.21–0.44)***
Unknown	265 (9.3)	29 (19.3)	1.06 (0.57–1.96)	76 (15.2)	0.82 (0.56–1.21)
Unemployment					
No	919 (32.4)	15 (10.0)	1	54 (10.8)	1
Yes	1917 (67.6)	135 (90.0)	4.32 (2.52–7.40)***	447 (89.2)	3.97 (2.96–5.32)***
Nursing home resident					
No	2746 (96.8)	137 (91.3)	1	455 (90.8)	1
Yes	90 (3.2)	13 (8.7)	2.90 (1.58–5.31)***	46 (9.2)	3.09 (2.13–4.46)***
Current smoking					
No	2278 (80.3)	129 (86.0)	1	419 (83.6)	1
1–10 cigarettes/day	189 (6.7)	9 (6.0)	0.84 (0.42–1.68)	30 (6.0)	0.86 (0.58–1.29)
>10 cigarettes/day	369 (13.0)	12 (8.0)	0.57 (0.32–1.05)	52 (10.4)	0.77 (0.56–1.04)
Any alcohol use					
No	2563 (90.4)	136 (90.7)	1	467 (93.2)	1
Yes	273 (9.6)	14 (9.3)	0.97 (0.55–1.70)	34 (6.8)	0.68 (0.47–0.99)*
ESRD receiving dialysis					
No	2794 (98.5)	148 (98.7)	1	474 (94.6)	1
Yes	42 (1.5)	2 (1.3)	0.90 (0.22–3.75)	27 (5.4)	3.79 (2.31–6.21)***
Malignancy					
No	2703 (95.3)	146 (97.3)	1	395 (78.8)	1
Yes	133 (4.7)	4 (2.7)	0.56 (0.20–1.53)	106 (21.2)	5.45 (4.14–7.19)***
TB history					
New case	2767 (97.6)	144 (96.0)	1	490 (97.8)	1
Relapse	69 (2.4)	6 (4.0)	1.67 (0.71–3.91)	11 (2.2)	0.90 (0.47–1.71)
AFB smear					
Negative	1438 (50.7)	45 (30.0)	1	241 (48.1)	1
Positive	1398 (49.3)	105 (70.0)	2.40 (1.68–3.43)***	260 (51.9)	1.11 (0.92–1.34)
Cavity on CXR					
No	2199 (77.5)	119 (79.3)	1	430 (85.8)	1
Yes	637 (22.5)	31 (20.7)	0.90 (0.60–1.35)	71 (14.2)	0.57 (0.44–0.74)***
Pleural effusion on CXR					
No	2676 (96.4)	139 (92.7)	1	440 (87.8)	1
Yes	160 (5.6)	11 (7.3)	1.32 (0.70–2.50)	61 (12.2)	2.32 (1.70–3.17)***
MDR-TB					
No	2811 (99.1)	148 (98.7)	1	496 (99.0)	1
Yes	25 (0.9)	2 (1.3)	1.52 (0.36–6.48)	5 (1.0)	1.13 (0.43–2.98)
Source of notification					
Outpatient services	1756 (61.9)	34 (22.7)	1	133 (26.5)	1
Ordinary ward	1028 (36.2)	100 (66.7)	5.02 (3.38–7.47)***	315 (62.9)	4.05 (3.26–5.03)***
Intensive care unit	52 (1.8)	16 (10.7)	15.89 (8.25–30.60)***	53 (10.6)	13.46 (8.83–20.51)***
Year of diagnosis					
2006	549 (19.4)	34 (22.7)	1	83 (16.6)	1
2007	621 (21.9)	32 (21.3)	0.83 (0.51–1.37)	115 (23.0)	1.23 (0.90–1.66)
2008	560 (19.7)	30 (20.0)	0.87 (0.52–1.43)	98 (19.6)	1.16 (0.85–1.59)
2009	546 (19.3)	28 (18.7)	0.83 (0.50–1.38)	92 (18.4)	1.12 (0.81–1.53)
2010	560 (19.7)	26 (17.3)	0.75 (0.44–1.27)	113 (22.6)	1.34 (0.98–1.81)

PTB, pulmonary tuberculosis; OR, odds ratio; CI, confidence interval; SAT, self-administered treatment; DOT, directly observed treatment; ESRD, end-stage renal disease; AFB, acid-fast bacilli; CXR, chest X ray; MDR-TB, multidrug-resistant tuberculosis;

* p<.05;

** p<.01;

*** p<.001.

† reference group.

Backward stepwise multinomial regression showed that, after controlling for other variables, patients on DOT had a 55% lower odds of TB-specific mortality (AOR = 0.45, 95% CI: 0.30–0.69) than those on SAT (Table 3). Prognostic factors associated with TB-specific and non-TB-specific mortality included age 65–79 and ≥80 years (reference: age 18–49 years), being unemployed, a positive AFB smear, and TB notification from a general ward or ICU (reference: outpatient services). Male sex, ESRD requiring dialysis, malignancy, and pleural effusion on chest radiography were significantly associated with non-TB-specific mortality, while presence of lung cavities on chest radiography was significantly associated with lower risk of non-TB-specific mortality.

**Table 3:** Multinomial regression: demographic and clinical variables associated with TB-specific and non-TB-specific deaths among adult PTB patients in Taipei, Taiwan, in 2006–2010†.

TB-specific deaths			Non-TB-specific deaths		
Factors	Crude OR (95% CI)	Adjusted OR (95% CI)	Factors	Crude OR (95% CI)	Adjusted OR (95% CI)
DOT	1.001 (0.70–1.43)	0.45 (0.30–0.69)***	DOT	1.36 (1.09–1.70)**	0.99 (0.76–1.29)
Age (years)			Age (years)		
18–49	1	1	18–49	1	1
50–64	1.85 (0.74–4.62)	1.72 (0.68–4.36)	50–64	3.95 (2.26–6.91)***	3.03 (1.70–5.41)***
65–79	4.66 (2.15–10.10)***	4.19 (1.87–9.40)***	65–79	10.33 (6.21–17.19)***	6.68 (3.91–11.42)***
≥80	15.75 (7.60–32.64)***	13.22 (6.11–28.57)***	≥80	20.77 (12.58–34.28)***	13.06 (7.70–22.16)***
-			Male gender	1.84 (1.46–2.32)***	1.39 (1.07–1.81)*
Unemployment	4.32 (2.52–7.40)***	1.84 (1.03–3.28)*	Unemployment	3.97 (2.96–5.32)***	1.42 (1.02–1.98)*
-	-	-	Malignancy	5.45 (4.14–7.19)***	4.76 (3.48–6.50)***
-	-	-	ESRD receiving dialysis	3.79 (2.31–6.21)***	2.80 (1.59–4.96)***
AFB positivity	2.40 (1.68–3.43)***	3.19 (2.11–4.82)***	AFB positivity	1.11 (0.92–1.34)	1.29 (1.02–1.63)*
-	-	-	Cavity on CXR	0.57 (0.44–0.74)***	0.72 (0.53–0.98)*
-	-	-	Pleural effusion on CXR	2.32 (1.70–3.17)***	1.73 (1.22–2.47)**
Source of notification			Source of notification		
Outpatient services	1	1	Outpatient services	1	1
Ordinary ward	5.02 (3.38–7.47)***	3.97 (2.62–6.00)***	Ordinary ward	4.05 (3.26–5.03)***	3.35 (2.64–4.24)***
Intensive care unit	15.89 (8.25–30.60)***	10.55 (5.30–21.01)***	Intensive care unit	13.46 (8.83–20.51)***	10.54 (6.64–16.75)***

† Reference is individuals with successful treatment.

PTB, pulmonary tuberculosis; OR, odds ratio; CI, confidence interval; DOT, directly observed treatment; AFB, acid-fast bacilli; ESRD, end-stage renal disease; CXR, chest X ray;

* p<.05;

** p<.01;

*** p<.001.

## Discussion

In this large cohort study of 3,487 Taiwanese adults with PTB, overall mortality was 18.7% in 2006–2010. After controlling for potential confounders, DOT was significantly associated with a lower risk of TB-specific mortality. Age older than 65 years, being unemployed, a positive AFB smear, and TB notification from a general ward or ICU were significantly associated with higher TB-specific and non-TB-specific mortality. Male sex, ESRD requiring dialysis, malignancy, and pleural effusion on chest radiography were predictors of non-TB-specific mortality, while presence of lung cavities on chest radiography was associated with a lower risk of non-TB-specific mortality.

As compared with TB patients in other Asian countries, TB mortality was higher in Taiwan than the 13.5% reported in South Korea [20] and 14.2% reported in Thailand [21]. The high mortality among PTB patients in these countries is a serious public health problem that deserves increased attention.

This study showed that DOT reduced TB-specific mortality after controlling for potential confounders. When the DOTS strategy was implemented in Taiwan in 2006, DOT was highly recommended as the optimal treatment strategy for patients with a high risk for transmitting infection [3]. Consequently, patients on DOT were significantly more likely than patients on SAT to have a positive sputum smear in this study. Previous studies also demonstrated that positive AFB smear was a risk factor for death among TB patients [22]–[24]. Hence, after controlling for AFB smear status and other covariates, the odds of TB-specific death among patients receiving DOT was 55% lower than those on SAT in this study.

In the Taipei DOTS program, DOT observers must have at least 9 years of education, attend a series of DOT training courses, and be qualified by the Taiwan CDC [3]. Each DOT observer monitors an average of 5 to 15 TB patients and was paid the equivalent of US$3/day for each case [3]. Under the supervision of public health nurses, DOT observers interview patients about their TB symptoms and treatment complications daily. When TB patients on DOT have, for example, worsened dyspnea or blurred vision, public health nurses will contact doctors to arrange a hospital visit. Public health nurses consult TB treatment guidelines daily to check the regimen for TB drugs for each DOT patient [25] and remind prescribing physicians to supply appropriate TB drugs to patients. The Taiwan CDC convenes a monthly TB expert panel to discuss complex cases (eg, multidrug-resistant TB patients) and give feedback to corresponding doctors [3]. To promote physician participation, doctors are given the equivalent of US$50 for every successfully treated TB patient [3]. The DOT experience in Taiwan shows that comprehensive interdisciplinary collaborations are needed if DOT programs are to be successful.

Our previous reports showed that DOT reduced all-cause mortality among TB patients in Taipei in 2006–2008 [19]. In the present study, the reduction in all-cause mortality was more likely due to the decrease in TB-specific mortality than from the decrease in non-TB-specific mortality. When mortality from TB-specific causes is very high, well-implemented DOT can reduce both TB-specific and all-cause mortality. However, when TB-specific mortality is not high, DOT, even when well implemented, might not significantly reduce all-cause mortality. Further research is needed to test this hypothesis.

Age older than 65 years was significantly associated with TB-specific and non-TB-specific mortality, perhaps because of immune system deterioration and the weakened physical condition of elderly adults [26]. Additionally, elderly patients with PTB are more likely to experience treatment delays because of nonspecific clinical presentations at TB onset (eg, no lung cavities on chest radiography) [27], [28]. Most of the study subjects were aged 65 years or older, and such patients require careful evaluation and monitoring during TB treatment.

Comorbidities such as malignancy and ESRD requiring dialysis were associated with non-TB-specific mortality in this study, perhaps because comorbidities predispose patients to more-severe disease [29] and lead to higher non-TB-specific mortality during treatment. The presence of a cavity on CXR, however, reduced the risk of non-TB-specific mortality among PTB patients in this study. This result suggests that cavity on CXR was a sign for TB diagnosis among culture-positive and culture-negative PTB patients [30], which might lead clinicians to initiate treatment early and hence reduce the complications of TB diseases [31]. Since 77.0% of deaths in this study resulted from non-TB-specific causes, comorbidities and additional health conditions should be screened in patients at the diagnosis of TB and if needed, a comprehensive treatment should be provided to reduce non-TB-specific mortality.

As in a previous study [23], we found that a positive AFB smear and being unemployed were associated with TB-specific mortality. However, MDR-TB, an established risk factor [23], was not significantly associated with TB-specific mortality in this report, perhaps because of the limited number of MDR-TB cases. Also, over two-third of MDRTB cases received DOT in this study, which might reduce the risk of TB-specific mortality.

Two limitations should be considered when interpreting the findings of this city-wide population-based study. First, due to our reliance on secondary data, important information about the TB patients, eg, data on diabetes, nutritional status and intravenous drug use, was not available in the surveillance data. Second, the patients were voluntarily enrolled in the DOT program. A prior study found that patients who were younger or of higher socioeconomic status were less likely to participate in the DOT program in Taipei [19]. In this study, mean age was 3 years older among patients on DOT than among those on SAT (p value<.001). Since older patients tended to have more comorbidities and higher mortality during treatment, this limitation might lead to underestimation of the effect of DOT on reducing TB-specific mortality, thereby reinforcing our main finding.

The strengths of this study include the fact that all eligible TB patients in Taipei were included in the analysis; thus, sample size was not based on considerations of statistical power. Another strength was the tightly regulated TB reporting system in Taiwan—cases of active pulmonary TB must be reported to local TB control departments within 24 hours and other TB cases no later than 7 days after diagnosis. Therefore, data were collected uniformly from patients, soon after diagnosis.

## Conclusions

We found that PTB patients on DOT had 55% lower TB-specific mortality than those on self-administered treatment, after covariate adjustment. We recommend that a comprehensive DOT program should be administered to all TB patients to further reduce TB-specific mortality in Taiwan.
